# Urothelial neoplasm in a 19‐year‐old male patient with urine discoloration, negative lab, and imaging workup: Should we investigate the findings or the symptom?

**DOI:** 10.1002/ccr3.1909

**Published:** 2019-01-17

**Authors:** Evangelos N. Symeonidis, Asterios Symeonidis, Chrysovalantis Gkekas, Christos Georgiadis, Apostolos Malioris, Michail Papathanasiou

**Affiliations:** ^1^ Department of Urology 424 General Military Hospital of Thessaloniki Thessaloniki Greece

**Keywords:** bladder, papillary, PUNLMP, TURBT, young

## Abstract

With few cases of PUNLMPs in young adults being reported in the literature, we hope to raise clinical awareness of prompt and effective diagnosis, while maintaining a high index of suspicion among health professionals. Even in the absence of red blood cells in the urine and subsequent negative imaging workup, clinicians should not delay performance of diagnostic cystoscopy.

## 1 INTRODUCTION

Urothelial tumors of the bladder represent a rare clinical entity in young adults <20 years old. Herein, we report a case of papillary urothelial neoplasm of low malignant potential (PUNLMP) in a 19‐year‐old young male. Interestingly, reddish discoloration of urine was the only clinical warning sign. Flexible diagnostic cystoscopy was performed followed by complete resection of the tumor.

## 2 CASE REPORT

**Figure 1:**
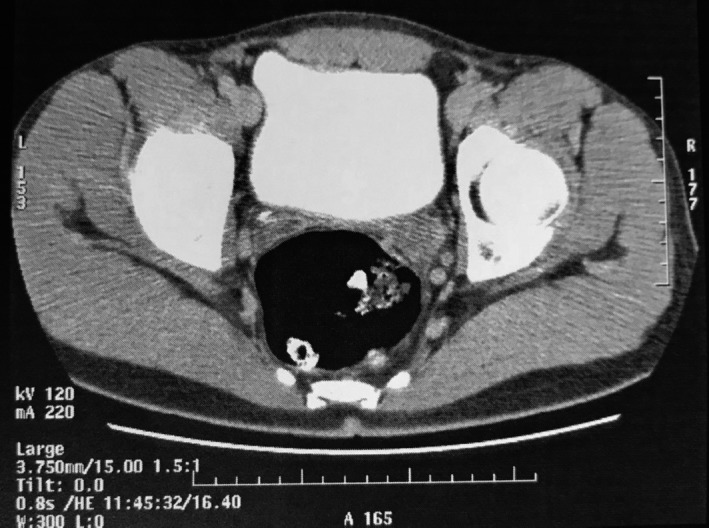
Axial view of first CT Urography (CTU), without any intravesical papillary mass or indication of filling defect

**Figure 2:**
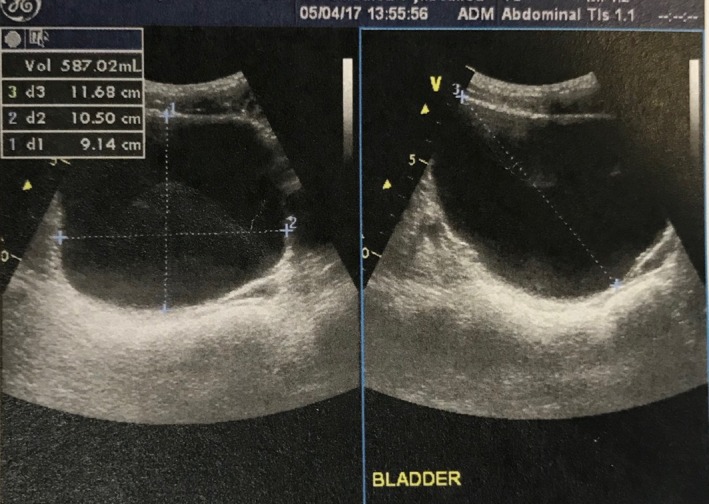
Image of transabdominal ultrasound of the bladder, failing to demonstrate intravesical papillary mass

**Figure 3:**
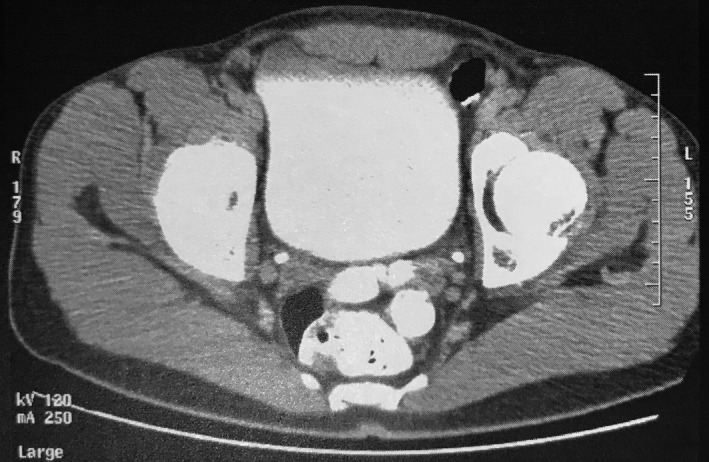
Axial view of second CT Urography (CTU) 6 mo later, indicating the absence of papillary lesion inside the bladder

Α 19‐year‐old healthy male presented to our urology department complaining about one single painless episode of reddish urine discoloration. No other symptoms or sexual intercourse were reported at that time. There was no family history of hereditary or other serious acquired diseases. Moreover, no significant predisposing risk factors for bladder cancer were identified. Initially, our patient consulted a private urologist and underwent a full blood count test, urinalysis, transabdominal ultrasound, and computed‐tomography urography (CTU). All laboratory tests were within normal limits and imaging modalities failed to indicate an intravesical papillary mass (Figures 1 and 2). A second similar episode of urine discoloration was reported, after a symptom‐free period of six months. Surprisingly, it was investigated one more time with CTU by a private urologist, again not significant for a pathologic filling defect (Figure 3).

Thereafter, our patient sought advice from our department and flexible cystoscopy was immediately performed. A well‐defined single papillary mass, approximately <10 mm in size, involving the dome of the bladder was identified. Transurethral resection of bladder tumor (TURBT)

was subsequently performed with excision of the papillary lesion. Histopathology revealed a papillary urothelial neoplasm of low malignant potential (PUNLMP) with minimal atypia in the mid‐to‐basal cell layers of the urothelium and prominent atypia of the superficial cells (so‐called “umbrella” cells). Some areas suggested an inverted component not regarded as an invasion.

Our patient recovered uneventfully and was suitable for discharge following day surgery with recommendations for follow‐up at three and six months. At 3‐ and 6‐month visits, cystoscopic findings were normal with no signs of recurrence. Follow‐up screening strategy included urine tests and flexible cystoscopy on a yearly basis for the next five years.

## 3 DISCUSSION

The majority of bladder cancers in young adults below the age of 20 years are low‐grade transitional cell carcinomas (TCCs) with a low recurrence rate.^1^, ^2^ The etiology differs from those above 40 years, mainly due to the lack of major risk factors for bladder cancer such as cigarette smoke, use of chemicals, bladder stones or infections. Given the early onset of disease, inherited genetic predisposition may underlie the current epidemic.^4^

It was not until recently that the Surveillance, Epidemiology and End Results (SEER) study conducted between 1973 and 2003 identified 140 cases of bladder tumors in children aged below 18 years. Papillary urothelial neoplasms of low malignant potential (PUNLMPs) comprised 50.7% of these tumors, with overall survival rates being reported as favorable.^1^

The most prominent clinical sign is painless visible hematuria.^2^, ^4^, ^5^ Imaging modalities such as ultrasound (US) and unenhanced computed tomography (CT) have been advocated with reliable results.^2^, ^5^, ^6^ Urine cytology has low detection rates due to the preponderance of low‐grade tumors; thus, it is not a useful tool.^4^, ^6^ On the contrary, cystoscopy remains the gold standard for diagnostic evaluation of hematuria and subsequent assessment of papillary intravesical masses.^3^, ^6^ There are cases, indeed, where urologists hesitate to perform a diagnostic cystoscopy. Delaying cystoscopy can be possibly explained by either lack of clinical awareness of bladder cancer in this particular age group or fear of causing penetrating urethral trauma.^4^, ^5^ Endoscopic resection via transurethral radical excision of the urothelial lesion is the standard of care, regardless of age.^4^, ^6^ Partial cystectomies have only been proposed for high‐grade tumors.^7^ A summary of the recent literature regarding the main published series of PUNLMPs can be found in Table 1.

**Table 1:** Review of main published series of PUNLMPs (last decade, 2007‐2018)

Author (Year)	Journal	Country	Period	Cases (Gender)	Age, mean, (range), y	Diagnostic Method	Symptom	Pathology	Type of surgery	Recurrence	Follow‐up, mean, (range), mo
Marinoni et al (2018)^9^	Bull Cancer	Italy	January 2006 to January 2016	2 (M)	11 (10‐12)	U/S	Incidental finding, hematuria	PUNLMP	TURBT	NO^a^	(6‐108)
Saltsman et al (2018)^10^	J Pediatr Surg	USA	January 1997 to September 2016	6 (1F/5M)	**19.2**	Cystoscopy	Hematuria/abdominal pelvic pain^a^	PUNLMP	TURBT	NO	**30.5**
Polat et al (2016)^8^	Int Braz J Urol	Turkey	2008‐2014	1 (M)	15	U/S	Hematuria	PUNLMP	TURBT	NO	24
Marte (2016)^11^	Austin J Urol	Italy	1990‐2014	5 (F)	13.2 (11‐15)	U/S, Cystoscopy	Gross hematuria, Incidental finding ‐ asymptomatic (1)	PUNLMP	TURBT (4), TURBT +iMMC (1)	NO	180
Berrettini et al (2015)^12^	J Pediatr Urol	Italy	January 1999 to July 2013	8	(5‐15)	U/S, CT Scan^a^	Incidental finding, hematuria^a^	PUNLMP	TURBT (7), TURBT +iMMC (1)	NO	60 (9‐174)^a^
Apoznanski et al (2015)^13^	Adv Clin Exp Med	Poland	1999‐2011	5 (M)	14.8 (7‐17)	U/S, Cystoscopy	Hematuria, Dysuria	PUNLMP	TURBT (3), TURBT +iDoxo (2)	NO	**48** (10‐120)^a^
Ander et al (2015)^14^	Int Urol Nephrol	Turkey	1980‐2014	1 (M)	12	U/S, IVU, Cystoscopy^a^	Abdominal pain	PUNLMP	TURBT	NO	26
Rifat et al (2015)^15^	Arab J Urol	Jordan	2009	1 (M)	5	n.a	Gross hematuria, interrupted urine stream	PUNLMP	TURBT	NO	36
Gao et al (2014)^5^	HK J Paediatr	China	n.a	1 (M)	9	U/S, unenhanced CT scan	Painless gross hematuria	PUNLMP	TURBT	NO	16
Alam et al (2007)^16^	Pediatr Transplant	USA	n.a	1 (F)	10	U/S, Cystoscopy	Incidental ‐ follow‐up after renal transplantation	PUNLMP	TURBT	NO	n.a

iDoxo: intravesical Doxorubicine; iMMC: intravesical mitomycin C; IVU: Intravenous urography; n.a: not available; PUNLMP: papillary urothelial neoplasm with low malignant potential; TURBT: transurethral resection of bladder tumor; U/S: Ultrasound.

Bold: median.

a Data not separate from other bladder neoplasms in the series.

Healthcare professionals need to be vigilant about every single episode of hematuria. Even in cases where urine reddish discoloration is the sole presenting symptom, as in our case, further investigation should not be omitted. Owing to low recurrence rates, even in low‐grade bladder tumors, close follow‐up is recommended.^4^ Of note, there is lack of comprehensive guidelines for long‐term monitoring of these patients; thus, several follow‐up time intervals have been proposed so far and various screening modalities.^2^, ^5^, ^6^ Bujons et al^3^ demonstrated follow‐up intervals ranging from 8 to 27 years. Polat et al^8^ proposed only postoperative ultrasound for screening, without the need of follow‐up cystoscopy.

Meanwhile, in the current body of literature dearth of data about the administration of adjuvant chemotherapy still exists.^7^ It is widely known that Bacillus Calmette‐Guérin (BCG) is used as an intravesical immunotherapy for treating early‐stage bladder cancer. Nevertheless, in patients below 20 years of age, the instillation of BCG remains questionable. Clinicians are hesitant to support the routine use of BCG instillations. Interestingly, Neogi et al^2^ reported the use of MVAC (methotrexate, vinblastine, adriamycin, cisplatin) in a 4‐year‐old boy, undergoing partial cystectomy for recurrence of high‐grade urothelial bladder carcinoma. Recently, Sheehan et al^4^ presented a case of intravesical instillation of mitomycin C following transurethral resection of bladder tumor (G2pTa TCCB) in an 18‐year‐old female patient. The development of a consistent and standardized surveillance protocol for urothelial neoplasms in this age group is of paramount importance.

## 4 CONCLUSION

Papillary urothelial neoplasms of low malignant potential (PUNLMP) are rare in young population. Various noninvasive diagnostic modalities have been introduced. Nevertheless, cystoscopy remains the mainstay of diagnosis for every episode of hematuria, while transurethral resection of tumor represents the treatment of choice. A high index of clinical suspicion is required for prompt and effective investigation even in cases of patient‐reported symptoms. Patient adherence to follow‐up plays a crucial role in diminishing recurrences and improving overall survival. Well‐designed epidemiological studies which will efficiently define the characteristics of urothelial neoplasms in young patients are warranted.

## INFORMED CONSENT

Written informed consent was obtained from the patient for publication of this case report and accompanying images.

## CONFLICT OF INTEREST

The authors have no conflict of interests to disclose.

## AUTHOR CONTRIBUTION

ENS: conceived and designed the study, acquired the data, analyzed and interpreted the data, wrote the manuscript, approved the final manuscript. AS, CG, CG, AM, MP: analyzed and interpreted the data, drafted the manuscript, approved the final manuscript.
